# Inbreeding effects on immune response in free-living song sparrows (*Melospiza melodia*)

**DOI:** 10.1098/rspb.2006.0092

**Published:** 2006-11-28

**Authors:** Jane M Reid, Peter Arcese, Lukas F Keller, Kyle H Elliott, Laura Sampson, Dennis Hasselquist

**Affiliations:** 1 School of Biological Sciences, Zoology Building, University of AberdeenTillydrone Avenue, Aberdeen AB24 2TZ, UK; 2 Centre for Applied Conservation Research, Forest Sciences, 2424 Main Mall, University of British ColumbiaVancouver BC, Canada V6T 1Z4; 3 Zoologisches Museum, Universität ZürichWinterthurerstrasse 190, 8057 Zürich, Switzerland; 4 Department of Zoology, University of ManitobaWinnipeg MB, Canada R3T 2N2; 5 Marine Biology Department, Centro Interdisciplinario de Ciencias Marinas, Instituto Politécnico NacionalCódigo Postal 23096, La Paz, BCS, México; 6 Department of Animal Ecology, Lund UniversityEcology Building, 223 62 Lund, Sweden

**Keywords:** cell-mediated immunity, conservation genetics, heterozygosity, humoral immunity, parasite-mediated selection

## Abstract

The consequences of inbreeding for host immunity to parasitic infection have broad implications for the evolutionary and dynamical impacts of parasites on populations where inbreeding occurs. To rigorously assess the magnitude and the prevalence of inbreeding effects on immunity, multiple components of host immune response should be related to inbreeding coefficient (*f*) in free-living individuals. We used a pedigreed, free-living population of song sparrows (*Melospiza melodia*) to test whether individual responses to widely used experimental immune challenges varied consistently with *f*. The patagial swelling response to phytohaemagglutinin declined markedly with *f* in both females and males in both 2002 and 2003, although overall inbreeding depression was greater in males. The primary antibody response to tetanus toxoid declined with *f* in females but not in males in both 2004 and 2005. Primary antibody responses to diphtheria toxoid were low but tended to decline with *f* in 2004. Overall inbreeding depression did not solely reflect particularly strong immune responses in outbred offspring of immigrant–native pairings or weak responses in highly inbred individuals. These data indicate substantial and apparently sex-specific inbreeding effects on immune response, implying that inbred hosts may be relatively susceptible to parasitic infection to differing degrees in males and females.

## 1. Introduction

The relationship between a host's inbreeding level and its immunity to parasitic infection is of broad interest in evolutionary and population ecology. A decline in immunity with inbreeding would indicate a dominance genetic component to parasite resistance, bearing on the evolutionary consequences of host–parasite interactions and the maintenance of genetic variation in natural populations (Potts & Wakeland 1990; Coltman *et al*. 1999; Westerdahl *et al*. 2005). Such declines would also suggest that parasites could mediate inbreeding depression in reproduction and survival, thereby threatening the persistence of small, inbred populations (Altizer *et al*. 2003; Tompkins *et al*. 2006; Whiteman *et al*. 2006). Quantifying the magnitude and the prevalence of inbreeding depression in immunity is therefore integral to predicting the genetic, evolutionary and population dynamic consequences of host–parasite interactions (Coltman *et al*. 1999; Reid *et al*. 2003; Spielman *et al*. 2004). Furthermore, in general, the evolutionary and dynamical impacts of parasites on host populations depend on whether the fitness cost of parasite exposure is constant or variable, and on the host sex, age or stage classes that are most affected (Murdoch *et al*. 1997; Coltman *et al*. 1999). Comprehensive studies of inbreeding effects on host immunity should therefore quantify whether effects act consistently or are exhibited only occasionally or in specific categories of population members (Giese & Hedrick 2003; Côté *et al*. 2005).

One common approach to measuring inbreeding effects on immunity is to relate host inbreeding level to the degree of infection or mortality after natural or experimental exposure to specific natural parasites (e.g. Cassinello *et al*. 2001; Haag *et al*. 2003; Spielman *et al*. 2004). A complementary approach is to relate inbreeding level to the host's response to a novel, experimental immune challenge (Giese & Hedrick 2003; Reid *et al*. 2003; Hawley *et al*. 2005). This approach has the advantage of eliminating confounding variation in a host's prior exposure to the focal parasite, thereby allowing inbreeding effects on immunity *per se* to be clearly distinguished from inbreeding effects on exposure (Norris & Evans 2000; Staszewski & Boulinier 2004). Such novel immune challenges are also ecologically and evolutionarily pertinent, since newly emerging parasites may exert particularly severe selection on naive host populations (Daszak *et al*. 2000; Altizer *et al*. 2003). Studies using this approach should apply multiple challenges targeting different components of host immunity, since different components may not show correlated responses (Norris & Evans 2000; Haag *et al*. 2003; Adamo 2004). Furthermore, since inbreeding effects on phenotypic traits are often less severe in captivity than in the wild (Hedrick & Kalinowski 2000; Joron & Brakefield 2003), studies should ideally be carried out in free-living individuals; the evolutionary and dynamical impacts of parasites on natural populations might otherwise be inaccurately inferred. However, despite their broad relevance, there remain few data describing relationships between host inbreeding *per se* and multiple components of immune response in free-living individuals, or the extent to which inbreeding effects vary among seasons or categories of population members.

We used a free-living, pedigreed population of song sparrows, *Melospiza melodia*, inhabiting Mandarte Island, Canada, to test whether individual responses to two widely used experimental immune challenges varied with an individual's coefficient of inbreeding (*f*). Specifically, we related individual variation in the patagial swelling response to phytohaemagglutinin (PHA), and in the humoral antibody response to diphtheria–tetanus vaccine (DTV), to individual *f*. We repeated each experimental challenge in two different years on different samples of males and females of varying ages, and thereby investigate whether inbreeding effects on immunity varied between years or with host age or sex.

## 2. Material and methods

### (a) Study population

Mandarte Island, approximately 6 ha in size, lies 25 km northeast of Victoria, British Columbia, Canada. Its resident song sparrow population, which numbered 16–27 breeding pairs during 2002–2005, has been studied intensively since 1975 (Smith *et al*. 2006). During this long-term study, all song sparrows fledged on Mandarte have been individually colour-ringed, almost always before leaving their natal territory. All immigrants to the breeding population (1.1 yr^−1^ on average) have been colour-ringed soon after settling. All population members are therefore individually identifiable (Smith *et al*. 2006). Detailed observations of breeding behaviour have allowed a complete social pedigree to be constructed, covering all sparrows hatched since 1981 (Keller 1998). We used standard algorithms to estimate each individual's *f* directly from the pedigree (Falconer & Mackay 1996; Keller 1998). The value of *f* reflects the probability that two homologous alleles will be identical by descent and estimates an individual's genome-wide homozygosity relative to the baseline population (Falconer & Mackay 1996). While immigrants to Mandarte are themselves of unknown *f*, they are genetically distinguishable from Mandarte's existing population at the time of arrival (assignment test based on nine microsatellite loci, *p*<0.02 for all immigrants during 1993–1996, see also Keller *et al*. 2001). Offspring of immigrant–native pairings can therefore be defined as outbred (*f*=0.000, Marr *et al*. 2002). Extra-pair fertilizations (epfs) occur on Mandarte (approx. 25% of offspring hatched during 1993–1996, O'Connor *et al*. 2006) and in song sparrows more widely (e.g. 11% of offspring, Major & Barber 2004). These epfs introduce error into the social pedigree and therefore into estimates of *f*. However, there is no evidence that epfs occur systematically with respect to relatedness in song sparrows on Mandarte. Briefly, across 283 broods hatched during 1993–1996, epfs were not more frequent in females that were more closely related to their social mates (*p*=0.52, O'Connor *et al*. in preparation). Furthermore, females were no more or less closely related to their extra-pair mate than to their social mate (*n*=100 triads, *p*=0.65, O'Connor *et al*. in preparation). Epfs are therefore likely to introduce error but not substantial bias into estimates of *f*. Such error is expected to cause inbreeding depression in phenotypic traits to be underestimated (Keller *et al*. 2002; Kruuk *et al*. 2002; Marr *et al*. in press). Since *f* reflects the relatedness between an individual's parents rather than an individual's relatedness to its own offspring, epfs will not necessarily introduce more error into estimates of *f* in males than in females. Such sex-biased error in *f*, and consequently in the estimated magnitude of inbreeding depression in phenotypic traits, could only arise given a biased sex ratio in extra-pair young. On Mandarte, the sex ratio of extra-pair young identified during 1993–1996 did not differ from 1 : 1 (65 males, 54 females, *χ*^2^_1_=0.4, *p*=0.53). Furthermore, sex ratio did not differ between within-pair and extra-pair young (*p*=0.61, Generalized linear mixed model controlling for year and brood). Therefore, although paternity of the song sparrows exposed to recent experimental immune challenges has not yet been fully verified, data from previous years do not lead us to expect sex-biased error in estimates of *f* or inbreeding depression.

### (b) PHA response

The patagial (wing-web) swelling response to subcutaneous injection of PHA is a widely used measure of avian immune responsiveness (Goto *et al*. 1978; Tella *et al*. 2002; Martin *et al*. 2006). PHA response is often interpreted as a measure of ‘cell-mediated immunity’, but may in fact reflect multiple immunological processes manifested as the infiltration and proliferation of multiple cell types over varying time periods (Martin *et al*. 2006). PHA response is thought to trade-off against other life-history components and to vary with individual condition and ‘quality’ (Tella *et al*. 2002; Martin *et al*. 2006), is often positively correlated with survival (Møller & Saino 2003) and has been related to MHC genotype (Taylor *et al*. 1987; Bonneaud *et al*. 2005). Therefore, while the underlying immunological processes may be more complex than often recognized, PHA response remains a useful measure of immunological ‘condition’ in the context of immunoecology.

During February 2002, September 2002 and September 2003, we measured PHA response in song sparrows on Mandarte. Methodology followed Reid *et al*. (2003). Briefly, sparrows were mist-netted after 11.00 and wing length, tarsus length and mass were recorded. Patagium thicknesses were measured three times using a modified dial calliper (Mitutoyo, Japan). Sparrows were injected with 30 μl 2 mg ml^−1^ PHA (L9132, Sigma, St Louis, Missouri) in phosphate buffer solution (PBS, 9.7 gl^−1^ D5773, Sigma, St Louis, Missouri) in the right patagium and 30 μl PBS in the left patagium and roosted overnight in individual enclosures with *ad libitum* food and water. Left and right patagial thicknesses were remeasured approximately 18 h after injection and sparrows were released. PHA response was estimated as the difference in increase in thickness between right and left patagia over the experimental period. Patagium thickness measurements were highly repeatable within individuals (*r*>0.94, *p*<0.0001, Reid *et al*. 2003).

### (c) Diphtheria–tetanus response

The specific humoral antibody response to foreign antigens constitutes one major component of avian acquired immunity (Roitt *et al*. 1998). During September 2004 and September 2005, we measured humoral immunity as a song sparrow's primary antibody response to tetanus and diphtheria toxoids (Hasselquist *et al*. 1999, 2001; Owen-Ashley *et al*. 2004). Sparrows were mist-netted, biometrics were recorded as above, and approximately 100 μl blood was collected by brachial venipuncture. Individuals were vaccinated with 70 μl human DTV(2 Lf diphtheria toxoid, 5 Lf tetanus toxoid adsorbed in aluminium phosphate, Aventis Pasteur Ltd, Toronto, Canada) in the pectoral muscle and released. Primary antibody responses peak 9–15 days after vaccination in song sparrows and other passerines (Hasselquist *et al*. 1999; Owen-Ashley *et al*. 2004). Correspondingly, we attempted to recapture sparrows 10–12 days after vaccination. Recaptured sparrows were reweighed, blood sampled as before and released. Since we could not control the exact timing of recapture of free-flying individuals and weather conditions impeded mist-netting on some days, we blood sampled all individuals recaptured 8–14 days after vaccination and subsequently controlled statistically for inter-sample period (see §2*d*). Blood samples were placed immediately on ice and centrifuged for 4 min at 3000rpm. within 5 h. Plasma was separated off, stored buried in ice and frozen at −20°C within 72 h. Enzyme-linked immunosorbent assays (ELISAs) were subsequently used to quantify tetanus and diphtheria antibody titres in pre- and post-vaccination plasma samples. Protocols followed those previously developed for song sparrows and other passerines (Hasselquist *et al*. 1999, 2001; Owen-Ashley *et al*. 2004). ELISA plates held an individual's pre- and post-vaccination plasma samples in duplicate. Titres were standardized for any among-plate variation in conditions or reagents within each year by reference to serially diluted standard samples that were included on each plate. Each individual's primary antibody response to tetanus and diphtheria toxoids was estimated as the difference between post- and pre-vaccination standardized antibody titres (Hasselquist *et al*. 1999; Owen-Ashley *et al*. 2004). Owing to within-year standardization of titres and slight between-year differences in ELISA assays, between-year differences in mean antibody response may not solely reflect true biological variation. In naive hosts, pre-vaccination antibody titres are expected to be low but non-zero due to natural antibodies that can bind antigens without previous exposure (Lee *et al*. 2006).

### (d) Analysis

We used general linear models (GLMs) to test whether PHA, tetanus or diphtheria responses varied with individual *f* within each year in which each immune challenge was applied, or across all data combined. We additionally modelled effects of individual age, body condition, maternal *f* and paternal *f* as covariates and sex and year as fixed factors. Sexes were determined by observing adult breeding behaviour or PCR amplification of sex-linked *CHD1* genes (Smith *et al*. 2006; Heinrich *et al*. in preparation). Body condition was estimated as the residual of mass on the cube of the first principal component of wing and tarsus length. Age was calculated to the nearest month from ringing data. We additionally modelled challenge date (within season) and inter-sample period as linear and quadratic covariates. Challenge date was eliminated from all models (all *p*>0.35). Some individuals vaccinated with DTV in 2005 were offspring of mothers that had been vaccinated in 2004. Since humoral immunity shows inter-generational effects of maternal vaccination in song sparrows (Reid *et al*. 2006), we included maternal vaccination history as a binary fixed factor (‘vaccinated’ or ‘unvaccinated’) in analyses of antibody responses in 2005. Since samples included sets of siblings, we initially modelled ‘family’ as a random factor. However, since family effects were not significant, models reduced to final GLMs.

A small number of individuals experienced the same immune challenge twice in consecutive years. Since repeat immune responses may not be independent (Roitt *et al*. 1998), we restricted analyses to each individual's first exposure to each challenge. We did not apply both PHA and DTV to the same individuals in the same year because mounting multiple simultaneous immune responses can reduce survival (Hansson *et al*. 2004). Ten individuals that experienced DTV during 2004–2005 had experienced PHA during 2002–2003. Diphtheria and tetanus responses did not differ between individuals that had and had not experienced PHA (*p*>0.6).

Since the Mandarte song sparrow pedigree is relatively deep, the only individuals that are considered completely outbred (*f*=0.000) are the offspring of immigrant–native pairings. Such F1 offspring of between-population crosses may show enhanced phenotypes due to heterosis (Falconer & Mackay 1996; Marr *et al*. 2002). Our samples also included some highly inbred offspring of pairings between first- or second-order relatives. Such pairings may involve a phenotypically non-random subset of parents (Reid *et al*. in preparation) and form relative outliers with respect to *f*. Therefore, to test whether overall declines in immune response with inbreeding were solely driven by heterosis in outbred offspring or by particularly weak responses in highly inbred individuals, we investigated whether final models or parameter estimates were altered by excluding outbred individuals, or individuals with *f*≥0.125.

Dependent variables were log transformed to reduce deviations from normality. Estimated slopes can consequently be directly interpreted as the inbreeding load or number of lethal equivalents (the slope of a log (trait) on *f* regression, Falconer & Mackay 1996). *B*_f_, *B*_m_ and *B* are the estimated inbreeding loads for females, males and all individuals (assuming no sex×*f* interaction) and are presented with 95% confidence limits. *η*^2^ is the partial effect size for *f*. Independent variables were not tightly correlated (all *r*<0.4). Residuals were approximately normally distributed and were not correlated or heteroscedastic with respect to predicted values. Immune response data were collected blind to individual *f* and other characteristics. Analyses were run in Pedigree Viewer (http://www-personal.une.edu.au/~bkinghor/pedigree.htm), SPSS (v. 14.0) and R (v. 2.2.1). All tests were two tailed. Variables were retained in models if *p*≤0.10. Two- and three-way interactions involving individual *f* were tested and eliminated except where stated. Sample sizes vary among models because body condition was not calculated for two individuals in wing moult during September 2002 and *f* was unknown for immigrant parents. Analyses of PHA response in 2002 differ from those reported previously (Reid *et al*. 2003) in that data from adult and juvenile sparrows are combined. Fieldwork was approved by the University of British Columbia Animal Care Committee.

## 3. Results

Table 1 describes the samples of song sparrows whose PHA, tetanus and diphtheria responses were measured during 2002–2005.

### (a) PHA response

Mean PHA response exceeded zero in both 2002 and 2003 (table 1). PHA response declined with increasing *f* in both 2002 and 2003 and across all data combined (table 2, figure 1). PHA response also increased with body condition in 2003 and across all data (table 2). There was also a significant sex×*f* interaction in 2003 and across all data; PHA response declined more markedly with *f* in males than in females (table 2). The final model remained quantitatively similar after excluding eight outbred individuals (*B*_f_=−2.1 (95% CI −5.3 to 1.0), *B*_m_=−4.3 (95% CI −5.8 to −2.8)). After excluding 12 highly inbred individuals, the sex×*f* interaction was no longer significant (*p*=0.47) but the estimated overall inbreeding load (*B*) increased to −4.3 (95% CI −5.7 to −2.9).

### (b) Tetanus response

Mean tetanus response was substantial in both 2004 and 2005 (table 1). Tetanus response declined with increasing *f* in 2004 and 2005 (assuming no sex×*f* interaction) and across all data (table 3). However, the main effect of sex and the sex×*f* interaction were significant in both 2004 and 2005 and across all data; females mounted slightly higher mean tetanus responses than males, and tetanus response declined with *f* in females but not in males (table 3, figure 2). Tetanus response also varied with inter-sample period and maternal vaccination history and tended to vary with paternal *f* in 2005. Sparrows whose mothers had been vaccinated in 2004 or whose fathers were relatively inbred showed stronger antibody responses (although the latter effect was weak, table 3, see also Reid *et al*. 2006). The final model remained quantitatively similar after excluding nine outbred individuals (*B*_f_=−7.4 (95% CI −12.8 to −2.0), *B*_m_=−1.5 (95% CI −5.0 to 2.0)) and qualitatively similar after excluding five highly inbred individuals (although confidence intervals around estimated inbreeding loads increased substantially in this case, *B*_f_=−11.2 (95% CI −20.5 to −1.9), *B*_m_=2.3 (95% CI −5.6 to 10.3)).

### (c) Diphtheria response

Mean diphtheria response exceeded zero in 2004 but not in 2005 (table 1). We therefore restricted analyses to 2004. Similarly low primary antibody responses to diphtheria toxoid have previously been reported in song sparrows (Owen-Ashley *et al*. 2004). Diphtheria and tetanus responses were correlated across individuals tested in 2004 (*N*=38, *r*=0.46, *p*=0.004).

Diphtheria response tended to decline with increasing *f* (figure 3) and increased then declined with age (age: *F*=18.2, *p*<0.001; age^2^: *F*=8.3, *p*=0.007). Females mounted marginally higher mean diphtheria responses than males (*F*=3.2, *p*=0.082), but the sex×*f* interaction was not significant (*p*=0.30). Other covariates were eliminated (all *p*>0.1; final model: *F*_4,37_=7.5, *p*<0.001, *R*^2^=0.41). The final model remained quantitatively similar after excluding a single outbred individual. After excluding four highly inbred individuals, the estimated inbreeding load increased to −4.4, although this effect no longer approached statistical significance due to increased confidence limits (95% CL −13.4 to 4.7, *p*=0.33).

## 4. Discussion

In free-living song sparrows, PHA, tetanus and diphtheria responses all varied to some extent with individual *f*. Estimated inbreeding loads for PHA and tetanus responses did not vary markedly among years or sparrows of different ages, but apparently differed between females and males. PHA response declined with *f* in both sexes, but declined more markedly in males than in females in 2003 and across all data. Tetanus response declined with *f* in females but not in males in 2004 and 2005 and across all data. We had sufficient statistical power to detect the inbreeding load for tetanus response estimated in females had it also occurred in males (observed *r*≈0.56, *n*=40, power≈0.97, given that overall variance in *f* was similar in both sexes). There is no clear expectation that apparent female-specific inbreeding depression should have arisen because measurement error in tetanus response or *f* was consistently greater in males (e.g. due to paternity error, see §2), and collinearity of explanatory variables did not differ between the sexes. Therefore, while it remains possible that the repeatable sex-specific inbreeding depression observed in tetanus response simply reflects sampling variance or stochastic male-biased error in *f*, our data suggest that tetanus response declined more markedly with *f* in females than males. Song sparrows showed weak primary diphtheria responses, especially in 2005 (see also Owen-Ashley *et al*. 2004). However, diphtheria response tended to decline with *f* across individuals tested in 2004. With respect to all the three measures of immune response, overall models were broadly robust to the exclusion of outbred and highly inbred individuals. Although the sex×*f* interaction no longer explained a significant proportion of variation in PHA response after excluding inbred individuals, and confidence intervals around parameter estimates were in some cases increased, overall patterns of declining immune responses with increasing *f* remained clear. Apparent inbreeding depression in immune response was therefore not solely attributable to heterosis in offspring of immigrants–native pairings, or to particularly weak immunity in the few highly inbred individuals that were tested.

Inbreeding depression is thought to reflect increased genetic homozygosity and consequently increased expression of deleterious recessive alleles and reduced expression of overdominance (Charlesworth & Charlesworth 1999). Inbreeding depression in immune response might therefore reflect direct effects of increased homozygosity at key response loci. However, since immune responses can be energetically demanding and depend on an individual's physiological and hormonal state (Hasselquist *et al*. 1999; Ots *et al*. 2001; Owen-Ashley *et al*. 2004), inbreeding depression may also reflect more general detrimental effects of inbreeding on metabolic or endocrinological pathways. PHA response declined with body condition in song sparrows, indicating condition dependence in this component of immunity (see also Alonso-Alvarez & Tella 2001; Råberg & Stjernman 2003). Tetanus and diphtheria responses did not vary with body condition (see also Råberg *et al*. 2003). However, the apparent sex-specific inbreeding depression observed in tetanus response may suggest an indirect physiological mechanism. Direct phenotypic consequences of inbreeding should not differ between females and males unless key loci are sex linked (Falconer & Mackay 1996), and major MHC loci that mediate major aspects of humoral (and cell-mediated) immunity are autosomal in birds (Edwards *et al*. 1999, although it is possible that sex-linked loci are also involved). In contrast, sex-specific variation in immune response resulting from sex-specific variation in life-history allocation and physiology is widely predicted and documented (Zuk & McKean 1998; Hasselquist *et al*. 1999; Stoehr & Kokko 2006). Predicted variation, however, primarily relates to mean responses and patterns of seasonal variation. The apparent sex-specific inbreeding depression observed in immune response is therefore unexpected. It is not clear why inbreeding effects should differ between males and females or why the direction of sex-specific effects should differ among components of immunity. Since investment in immune responsiveness may trade-off against investment in other life-history components (Tella *et al*. 2002; Adamo 2004), one final possibility is that relatively inbred individuals strategically invest less in immunity. Such reduced allocation could be viewed in a life-history context as an adaptive response to increased intrinsic mortality rates in inbred individuals, and could conceivably be modulated in response to direct physiological or endocrinological effects of inbreeding. Such strategic investment patterns may provide one possible explanation for sex-specific allocation to immunity with respect to *f*.

Irrespective of the underlying mechanisms, our results broadly concur with an increasing body of evidence that parasite resistance commonly declines with reduced heterozygosity and/or genetic diversity in vertebrates (e.g. Acevedo-Whitehouse *et al*. 2005; MacDougall-Shackleton *et al*. 2005; Westerdahl *et al*. 2005; Whiteman *et al*. 2006, although see Giese & Hedrick 2003; Côté *et al*. 2005). However, most studies, particularly those on free-living individuals, have related parasite resistance or immunity to heterozygosity or genetic diversity measured across a few microsatellite or MHC loci rather than individual *f*. In many cases, it is therefore uncertain whether observed effects reflect genome-wide heterozygosity or inbreeding *per se*, as opposed to heterozygosity at specific focal or linked loci (Balloux *et al*. 2004; Slate *et al*. 2004). Furthermore, few studies have related any measure of inbreeding, heterozygosity or genetic diversity to an individual's response to any novel, non-specific immune challenge, and no general pattern has emerged. For example, one generation of sib–sib mating did not reduce encapsulation response in bumble-bees (*Bombus terrestris*, Gerloff *et al*. 2003). PHA response but not the antibody response to sheep red blood cells (SRBC response) increased with multilocus heterozygosity in captive house finches (*Carpodacus mexicanus*, Hawley *et al*. 2005). Neither PHA nor SRBC response varied with MHC diversity in captive house sparrows (*Passer domesticus*, Bonneaud *et al*. 2005). In contrast, PHA, tetanus and diphtheria responses all declined to some extent with increasing *f* in song sparrows. Further studies are required to characterize the patterns and causes of this variation and identify the circumstances under which inbreeding and/or genetic diversity is most likely to influence immune response.

It is likely to be simplistic to interpret an individual's response to any single immune challenge as a measure of general parasite resistance (Norris & Evans 2000; Adamo 2004). Since only ten song sparrows experienced both PHA and DTV and these individuals experienced these challenges in different years, we have little power to assess whether PHA, diphtheria and tetanus responses were correlated within individuals. Notwithstanding these limitations, individual PHA and tetanus responses tended to be negatively correlated after controlling for year, inter-sample period and maternal vaccination history (*r*_s_=−0.55, *n*=10, *p*=0.09). However, our repeated challenge experiments suggested that PHA, tetanus and diphtheria responses, which to some degree reflect the main components of avian immunity (Norris & Evans 2000; Martin *et al*. 2006), show broadly similar declines with increasing *f* in free-living song sparrows. Inbreeding loads were estimated as 2.1–7.0, 7.2–7.8 and 2.8 lethal equivalents per gamete for PHA, tetanus (in females) and diphtheria responses, respectively. Individual *f* explained a remarkably high proportion of variation, particularly in PHA response. Furthermore, given the likely paternity error in the Mandarte pedigree, these figures may underestimate the true magnitude of inbreeding depression (Keller *et al*. 2002; Kruuk *et al*. 2002; Marr *et al*. in press). We cannot quantitatively compare the inbreeding loads observed in PHA, diphtheria and tetanus responses, since these different components of immunity were measured in different individuals in different years. Among-component variation in inbreeding depression cannot therefore be distinguished from among-year variation, such as might result from among-year variation in environmental severity (e.g. Keller *et al*. 2002). However, our data indicate that the magnitude of inbreeding depression can be of the same order in multiple components of immunity, at least in females, and equals or exceeds that commonly observed in major fitness components, including in song sparrows on Mandarte (Keller 1998; Kruuk *et al*. 2002).

The substantial inbreeding depression observed in immune response seems likely to be biologically significant. Variation in PHA response has been linked to substantial changes in life-history allocation (Martin *et al*. 2006), and an average effect size of *r*=0.43 has been reported for the relationship between immune response and survival in birds (Møller & Saino 2003; see also Råberg & Stjernman 2003). Given this effect size and the inbreeding depression in immunity observed in song sparrows, even moderate inbreeding (*f*≈0.1) would equate to a substantial reduction in adult survival. Therefore, interpreted directly, our data indicate that even moderate inbreeding may impair immunity sufficiently to reduce fitness in free-living birds (assuming exposure to a relevant parasite and notwithstanding that fitness may not be maximized by maximizing immune responsiveness, Råberg & Stjernman 2003; Adamo 2004; Viney *et al*. 2005). These data support the general suggestion that host–parasite interactions can be influenced by host genotype and specifically highlight the likely importance of dominance genetic effects such that the parasite resistance declines with host genome-wide heterozygosity. Resulting parasite-mediated selection against inbred hosts may help maintain genetic diversity in natural populations (Potts & Wakeland 1990; Coltman *et al*. 1999), influence the evolution and maintenance of inter-sexual selection (Reid *et al*. 2005) and threaten the persistence of small populations (Altizer *et al*. 2003; Whiteman *et al*. 2006). The apparent sex-specific inbreeding depression observed in PHA response and particularly tetanus response is unexpected, and implies that further compound consequences of host–parasite interactions might arise. For example, given sex-specific inbreeding depression in immunity, parasites might cause biased sex ratios in small populations of inbred hosts, thereby reducing effective population size and further increasing inbreeding, genetic drift and stochastic extinction risk. Further studies are required to investigate the prevalence, magnitude and causes of sex-specific inbreeding depression in parasite resistance and explore the demographic and evolutionary implications.

## Figures and Tables

**Figure 1:**
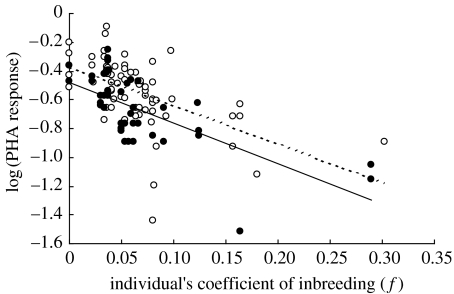
Relationships between an individual song sparrow's coefficient of inbreeding (*f*) and (log) phytohaemagglutinin (PHA) response measured in 2002 (open symbols, dashed line) and 2003 (filled symbols, solid line).

**Figure 2:**
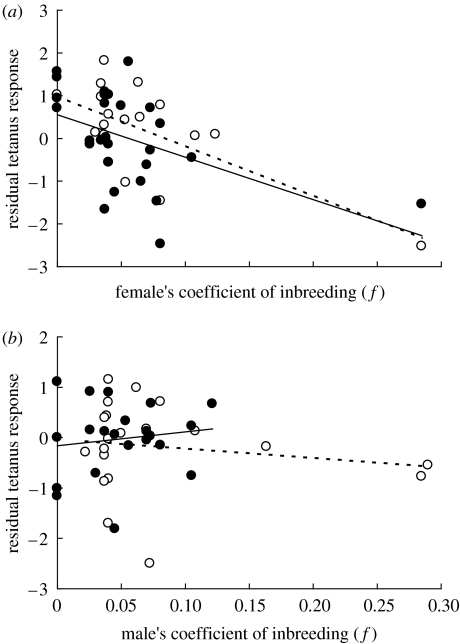
Relationships between an individual song sparrow's coefficient of inbreeding (*f*) and residual (log) tetanus response (controlling for inter-sample period, maternal vaccination history and paternal *f*) for (*a*) females and (*b*) males, measured in 2004 (open symbols, dashed line) and 2005 (filled symbols, solid line).

**Figure 3:**
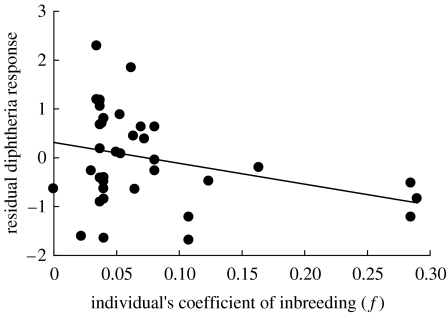
Relationship between an individual song sparrow's coefficient of inbreeding (*f*) and residual (log) diphtheria response (controlling for age and sex) measured in 2004. Diphtheria response tended to decline with *f* (*F*=3.7, *p*=0.065, *B*=−2.8 (95% CL −5.8 to 0.2), *η*^2^=0.10).

**Table 1:** Descriptive statistics of song sparrows challenged with phytohaemagglutinin (PHA) and diphtheria–tetanus vaccine (DTV) during 2002–2005. (*N*_t_, *N*_juv_ and *N*_ad_ are the total samples sizes of naive individuals, juveniles (individuals hatched in the current year) and adults (individuals hatched in previous years). Ranges are shown in parentheses. Means are shown ±1s.e. Mist-netting effort totalled approximately 26 000 net metre hours.)

test	year	*N*_t_	*N*_juv_	*N*_ad_	median *f*	median age of adults (months)	mean inter-sample period (days)	mean immune response	difference of mean response from zero
PHA	2002	72	35	37	0.053	16	—	0.32±0.02	*t*_1,71_=19.1, *p*<0.001
(0.000–0.303)	(9–93)			
PHA	2003	44	37	7	0.052	52	—	0.25±0.02	*t*_1,43_=13.6, *p*<0.001
(0.000–0.289)	(16–88)			
DTV	2004	38	26	12	0.04	16	9.7±0.3	Tet: 76.7±14.6	*t*_1,37_=5.2, *p*<0.001
(0.000–0.289)	(16–52)	(8–14)	Dip: 13.2±4.3	*t*_1,37_=3.0, *p*=0.004
DTV	2005	48	46	2	0.04	—	9.5±0.2	Tet: 235.1±38.0	*t*_1,47_=6.2, *p*<0.001
(0.000–0.285)	(40&52)	(8–13)	Dip: 4.1±2.3	*t*_1,47_=1.7, *p*=0.090

**Table 2:** Models explaining variation in PHA response in song sparrows. (*N*_t_, *N*_f_ and *N*_m_ are the total sample sizes of naive individuals, females and males included in final models. Variables retained in final models are indicated in bold. Age×*f*, year×*f* and year×sex×*f* interactions were not significant (*p*>0.20).)

year	*N*_t_	*N*_f_	*N*_m_	individual *f*	age	body condition	maternal *f*	paternal *f*	sex	sex×*f*	season or year	final model
2002	72	29	43	***F***=**29.4**	*F*=1.7	*F*=1.7	*F*=0.04	*F*=0.5	*F*=0.4	*F*=0.8	*F*=0.8	*F*_1,71_=29.4
				***p***<**0.001**	*p*=0.20	*p*=0.19	*p*=0.85	*p*=0.48	*p*=0.55	*p*=0.78	*p*=0.37	*p*<0.001
				*B*=−2.7 (−3.6–−1.7)								*R*^2^=0.29
*η*^2^=0.29								
2003	44	21	23	***F***=**64.9**	*F*=0.03	***F***=**5.1**	*F*=0.01	*F*=0.1	***F***=**7.0**	***F***=**19.4**	—	*F*_4,43_=22.9
				***p***<**0.001**	*p*=0.87	***p***=**0.029**	*p*=0.93	*p*=0.70	***p***=**0.012**	***p***<**0.001**		*p*<0.001
				*B*_f_=−2.1 (−4.4–−0.1)								*R*^2^=0.67
				*B*_m_=−7.0 (−9.1–−4.9)								
				*B*=−2.7 (−3.7–−1.8)								
*η*^2^=0.63								
all data	114	49	65	***F***=**71.5**	*F*=0.7	***F***=**9.3**	*F*=0.1	*F*=0.8	***F***=**0.4**	***F***=**4.8**	***F***=**9.7**	*F*_6,113_=16.8
				***p***<**0.001**	*p*=0.42	***p***=**0.003**	*p*=0.74	*p*=0.37	***p***=**0.52**	***p***=**0.030**	***p***<**0.001**	*p*<0.001
				*B*_f_=−2.3 (−3.8–−0.8)								*R*^2^=0.46
				*B*_m_=−3.9 (−5.2–−2.7)								
				*B*=−2.8 (−3.4–−2.1)								
*η*^2^=0.40								

**Table 3:** Models explaining variation in tetanus response in song sparrows. (*N*_t_, *N*_f_ and *N*_m_ are the total sample sizes of naive individuals, females and males included in final models. There was insufficient variance to include age as an explanatory variable in 2005 (table 1). Variables retained in final models are indicated in bold. Age×*f*, year×*f* and year×sex×*f* interactions were not significant (*p*>0.35).)

year	*N*_t_	*N*_f_	*N*_m_	individual *f*	inter-sample period	inter-sample period^2^	age	body condition	maternal *f*	paternal *f*	sex	sex×*f*	maternal vaccination history	year	final model
2004	38	18	20	***F***=**9.9**	***F***=**7.0**	***F***=**5.0**	*F*=2.4	*F*=0.2	*F*=0.01	*F*=0.2	***F***=**5.5**	***F***=**4.9**	—	—	*F*_5,37_=5.2
***p***=**0.003**				***p***=**0.013**	***p***=**0.033**	*p*=0.13	*p*=0.66	*p*=0.96	*p*=0.62	***p***=**0.025**	***p***=**0.033**			*p*=0.001
*B*_*f*_=−7.8 (−13.7–−2.0)														*R*^2^=0.36
*B*_m_=−1.5 (−5.0–2.1)														
*B*=−3.8 (−6.8–−0.7)														
*η*^2^=0.24											
2005	47	27	20	***F***=**0.5**	***F***=**6.1**	***F***=**5.9**	—	*F*=0.01	*F*=0.10	***F***=**3.5**	***F***=**3.3**	***F***=**4.4**	***F***=**7.8**	—	*F*_7,46_=5.5
				***p***=**0.49**	***p***=**0.018**	***p***=**0.020**		*p*=0.93	*p*=0.77	***p***=**0.071**	***p***=**0.076**	***p***=**0.044**	***p***=**0.008**		*p*<0.001
				*B*_*f*_=−7.2 (−14.9–−0.3)											*R*^2^=0.41
				*B*_m_=3.8 (−5.3–12.9)											
				*B*=−4.7 (−8.8–−0.5)											
*η*^2^=0.12											
all data	85	45	40	***F***=**14.9**	***F***=**11.1**	***F***=**8.8**	*F*=2.4	*F*=1.1	*F*=0.2	***F***=**2.8**	***F***=**4.8**	***F***=**7.5**	***F***=**11.4**	***F***=**37.2**	*F*_8,84_=20.8
				***p*****<0.001**	***p***=**0.001**	***p***=**0.004**	*p*=0.13	*p*=0.29	*p*=0.67	***p***=**0.10**	***p***=**0.031**	***p***=**0.008**	***p***=**0.001**	***p*****<0.001**	*p*<0.001
				*B*_f_=−7.7 (−12.4–−3.0)											*R*^2^=0.65
				*B*_m_=−1.1 (−4.5–2.3)											
				*B*=−4.5 (−7.1–−2.2)											
*η*^2^=0.16											
